# The Use of Intervention Mapping to Develop a Tailored Web-Based Intervention, Condom-HIM

**DOI:** 10.2196/publichealth.7052

**Published:** 2017-04-19

**Authors:** Joyal Miranda, José Côté

**Affiliations:** ^1^ Faculty of Community Services Daphne Cockwell School of Nursing Ryerson University Toronto, ON Canada; ^2^ Faculté des sciences infirmières Université de Montréal Montreal, ON Canada

**Keywords:** Internet, condoms, HIV seropositivity, intention, self-efficacy, sexual behavior

## Abstract

**Background:**

Many HIV (human immunodeficiency virus) prevention interventions are currently being implemented and evaluated, with little information published on their development. A framework highlighting the method of development of an intervention can be used by others wanting to replicate interventions or develop similar interventions to suit other contexts and settings. It provides researchers with a comprehensive development process of the intervention.

**Objective:**

The objective of this paper was to describe how a systematic approach, intervention mapping, was used to develop a tailored Web-based intervention to increase condom use among HIV-positive men who have sex with men.

**Methods:**

The intervention was developed in consultation with a multidisciplinary team composed of academic researchers, community members, Web designers, and the target population. Intervention mapping involved a systematic process of 6 steps: (1) needs assessment; (2) identification of proximal intervention objectives; (3) selection of theory-based intervention methods and practical strategies; (4) development of intervention components and materials; (5) adoption, implementation, and maintenance; and (6) evaluation planning.

**Results:**

The application of intervention mapping resulted in the development of a tailored Web-based intervention for HIV-positive men who have sex with men, called Condom-HIM.

**Conclusions:**

Using intervention mapping as a systematic process to develop interventions is a feasible approach that specifically integrates the use of theory and empirical findings. Outlining the process used to develop a particular intervention provides clarification on the conceptual use of experimental interventions in addition to potentially identifying reasons for intervention failures.

## Introduction

Recent studies have found that individuals who use the Internet to meet sexual partners are at an elevated risk of contracting human immunodeficiency virus (HIV) infection and other sexually transmitted diseases (STDs) [1-3]. Individuals who seek partners via the Internet tend to engage in riskier sexual behavior (ie, a seropositive man having insertive condomless anal sex with a partner who is seronegative or whose serostatus is unknown) [4-6]. Several researchers studying the population of men who have sex with men (MSM) have identified a significant association between online partner seeking and an increased incidence of HIV transmission [2,5] and have speculated that using the Internet to seek sexual partners may be fueling HIV risk behaviors within this group [7].

Using the Internet as a method of meeting sexual partners came to the attention of public health officials after a syphilis outbreak in San Francisco in 1999 was associated with increasing transmission among MSM, who were HIV-positive and also met their partners online [8]. Since then, other studies have also examined the transmission of HIV and other STDs among MSM who met online [7,9,10]. In particular, a meta-analysis involving Internet-using MSM found that men who seek sexual partners via the Internet, especially HIV-positive men, were more likely than men who did not seek sexual partners on the Internet to engage in unprotected anal intercourse [7]. For many, high-risk sexual behaviors continued after HIV diagnosis. The reductions seen in high-risk behaviors were short-lived, and the individuals tended to revert to engaging in HIV transmission high-risk behaviors within 1 year of diagnosis [11]. From a public health perspective, efforts to prevent HIV-positive, Internet-using MSM from transmitting HIV have been focused on the sexual high-risk behavior of having unprotected insertive and receptive anal sex with HIV-negative partners and those of unknown serostatus. As MSM were early adopters of the Internet, and given the increase in high-risk sexual behaviors, strategies using the same method of socializing should be considered when interventions are developed to prevent HIV transmission among this particular target population [12].

To date, only a small number of studies have been reported on the efficacy of Internet-based interventions targeting HIV-positive MSM in influencing risky behaviors such as inconsistent condom use [13,14]. Moreover, many of these have neglected to address the process by which the intervention was developed. Systematically developing an intervention based on empirical evidence and theory has been found to substantially improve the chances of the intervention’s success, as well as of identifying possible causes for its failures [15]. Lack of information on the systematic development and content of an intervention poses limits not only to adequate intervention evaluation, but also to any meta-analytic reviews of the intervention’s effectiveness that may be conducted in the future [16]. The purpose of this paper is to describe the systematic process followed to develop a tailored Web-based intervention, Condom-HIM. In this case, intervention mapping was used to design an intervention aimed at increasing condom use among HIV-positive MSM who do not consistently use condoms with their partners who are either HIV-negative or of unknown serostatus.

## Methods

**Figure 1:**
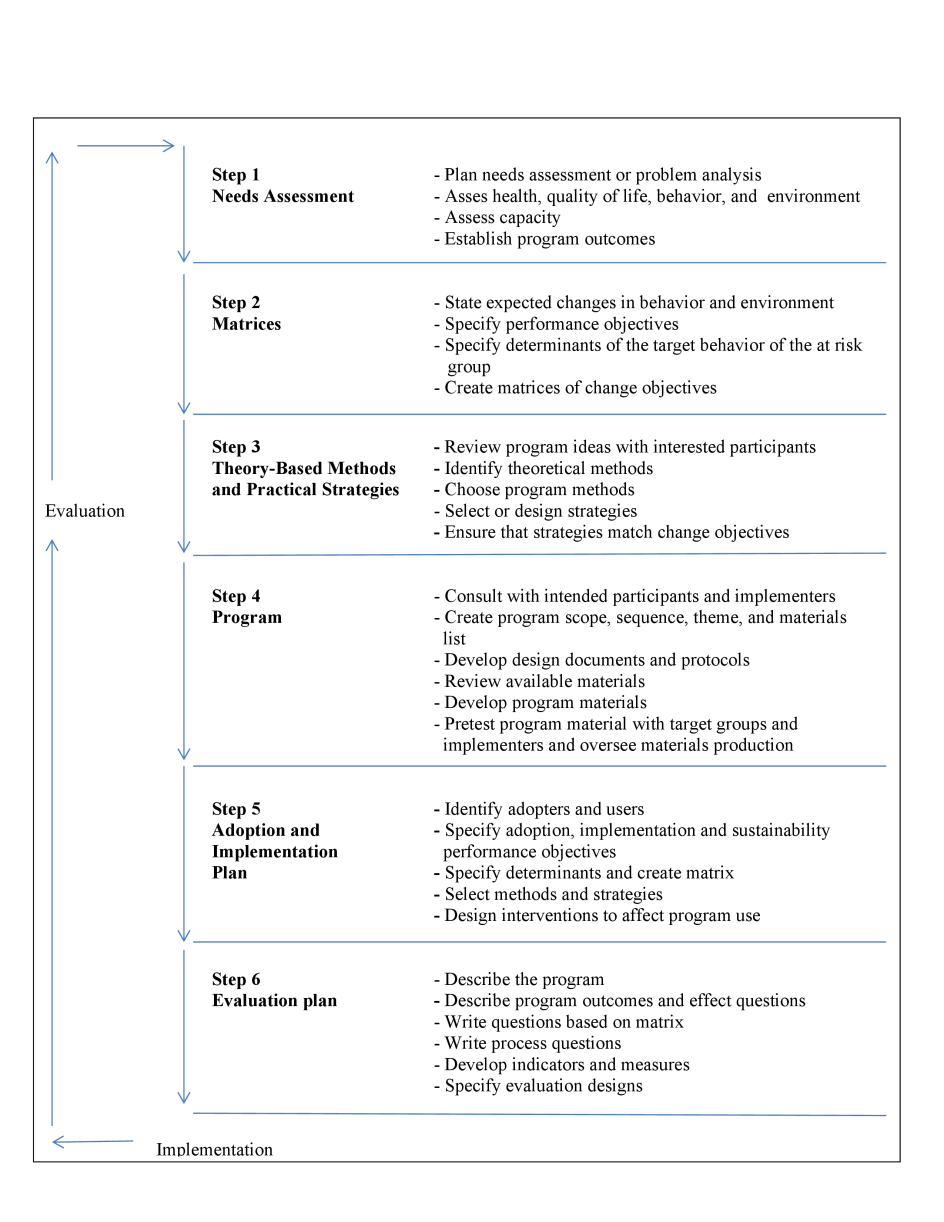
The intervention mapping process.

Intervention mapping, the systematic process applied to develop the tailored Web-based intervention, is composed of the following 6 steps (see Figure 1) that build on each other: (1) conducting a needs assessment; (2) creating a detailed map of intervention objectives and the behavioral and environmental determinants of the problem that the intervention would address; (3) selecting theory-based methods and practical strategies to modify the behavioral and environmental determinants; (4) producing the intervention’s components and materials; (5) planning for adoption, implementation, and sustainability; and (6) creating evaluation plans and instruments [17]. The intervention mapping process allows the intervention’s developers to make effective decisions at each step in its development based on empirical evidence, theory, and information collected from the target population. In this paper, the first 4 steps of the intervention mapping process and the particular tasks carried out within them are described in detail. Steps 5 and 6, which involve intervention implementation, adoption, monitoring, and evaluation, are only briefly highlighted because the paper’s focus is on the development of the intervention.

## Results

### Development Team

A multidisciplinary research team was first established in order to develop the intervention using the various steps of the intervention mapping process. Individuals were selected to represent various fields of expertise that were required for the intervention’s design, and such individuals included the following:

- a behavioral researcher with expertise in adopting, maintaining, and changing health-related behaviors such as condom use, as well as in Web-based intervention design and delivery;
- two sexologist researchers with a focus on psychosocial and sociocultural factors associated with at-risk or preventive behaviors, particularly in relation to STDs within the MSM population;
- two nursing researchers with expertise in the field of HIV and AIDS, as well as delivery and development of Web-based interventions;
- one computer and software research engineer;
- three sexual health community-based clinicians with access to the target population;
- individuals representing the target population;
- a Web design team to support the visual development of the Web-based intervention.

### Intervention Mapping Step 1: Needs Assessment

The first intervention mapping step of conducting a needs assessment helps to identify the health problem and its related behavioral and environmental determinants for the at-risk population [18]. A call was put out by one of the local sexual health community centers for HIV-positive MSM willing to participate in interviews asking about their needs in relation to HIV and AIDS prevention issues. The principal investigator of the study conducted a total of 10 interviews with HIV-positive MSM from the local sexual health community center as well as 2 interviews with HIV counselors from the center who also fit into the target population in order to identify the current needs of MSM. It was identified by the target population that there was indeed a need to have more innovative and interactive support through the use of the Internet in relation to condom use, while still allowing for anonymity of their HIV status. The current methods to date in relation to increasing condom use required interventions that were face-to-face, thereby requiring individuals to identify their HIV status if wanting to participate. In addition to the needs assessment, the multidisciplinary team also met to discuss the prevalent issues within the community and review the empirical literature. They found that, of the groups within Canada considered most at risk for contracting HIV, MSM accounted for the greatest proportion (54.3%) of new HIV infections in 2014 [19]. One main source of transmission identified was unsafe sex among people who know they are infected with HIV [20]. Various studies targeting the sexual risk behaviors in MSM living with HIV have found that 10%-60% do not consistently practice safer sex behaviors [20]. In addition, MSM recently infected with HIV reported that they had a median of 20 sexual partners within the previous year and that they continued to repeatedly engage in high-risk sexual behaviors, specifically unprotected anal intercourse [11]. Of particular interest is the fact that 34% of MSM with recently diagnosed HIV did not change their risk behaviors following diagnosis, and 20% actually increased their risk behaviors after diagnosis [11]. Such potential results of engaging in unprotected anal sex results in putting oneself at risk of contracting secondary infections (eg, syphilis, gonorrhea, and herpesvirus), which in turn have the potential to accelerate the HIV disease [20,21].

Evidence has indicated that 35%-45% of MSM access the Internet to seek sexual partners [4]. Furthermore, this group of MSM was more likely to report unprotected anal intercourse with nonconcordant casual partners they met online [3]. Halkitis and Parsons [22] found that 84% of MSM reported engaging in unprotected anal intercourse in the past 3 months and that 43% of the men reported recent unprotected anal intercourse with a partner of unknown serostatus. These results indicated the potential for widespread transmission of HIV to uninfected men by the partners they met on the Internet.

On the basis of the needs assessment and review of the literature, a Web-based intervention was considered to be potentially useful in targeting HIV-positive MSM seeking partners online. Although most studies targeted HIV-negative or untested individuals, both the National Institutes of Health and the Centers for Disease Control and Prevention have proclaimed the need for behavioral interventions directed toward individuals living with HIV [23]. Prevention interventions are needed to help HIV-positive MSM adopt and maintain safer sex behaviors such as condom use. Without such interventions, the growing number of MSM who use the Internet to seek sexual partners will continue to form a potential source of HIV transmission to HIV-negative MSM or those of unknown status [24]. Preventing new infections has represented the only long-term, sustainable way to curb the spread of HIV and AIDS [25]. Consequently, the research team reviewing the empirical evidence in relation to decreasing high-risk behaviors with HIV-positive MSM who use the Internet to seek sexual partners identified unprotected anal intercourse as the relevant risk behavior within this population.

### Intervention Mapping Step 2

#### Matrices of Change Objectives

The second step of intervention mapping provides the foundation for the intervention’s design by specifying who and what will change as a result of the intervention. A set of matrices was generated by the research team in order to identify the intervention’s performance objectives, which is what the at-risk group members must do to accomplish the health-related behavior of HIV-positive MSM to consistently and correctly use condoms for anal sex with their partners who were either HIV-negative or of unknown serostatus.

Condom use was chosen as the behavioral outcome over other forms of HIV prevention such as serosorting and viral load status. Serosorting is defined as the practice of having sex with partners of concordant HIV status. This form of HIV prevention has been practiced by many HIV-positive MSM. Research has shown, however, that serosorting is unlikely to be beneficial in many populations of MSM and can actually be expected to lead to increased risk of HIV transmission [26]. In a systematic review to assess the association between serosorting and HIV conducted among MSM, it was found that compared with condom use serosorting was associated with a higher risk of HIV (odds ratio, OR, 1.80, 95% CI 1.21-2.70) [27].

Results of studies regarding viral loads have shown that, since the introduction of antiretroviral therapy, MSM have perceived that having an undetectable HIV load reduces the risk of HIV transmission [28-31]. For instance, Lampe [28] found that, among MSM receiving antiretroviral therapy, those who reported undetectable viral load had higher rates of condomless sex with a partner of different serostatus than those who did not. In addition, a recent study by Van Den Boom et al [29] found that, among MSM, between 20% and 57% of reported practices of engaging in unprotected anal sex were related to having an undetectable viral load. Also of interest was that, among HIV-discordant partners, undetectable viral loads were considered before engaging in unprotected anal sex with sex buddies (40% of the time) and with casual partners (57% of the time). The problem with this particular strategy of prevention is that, even if the viral load is undetectable, unknown risk parameters, viral load variability, and the possibility of drug-resistant strains of HIV still makes this strategy an unreliable and inconsistent way to prevent HIV transmission [32].

#### Performance Objectives

In order to specifically target the behavioral change among MSM, the research team next identified performance objectives that would clarify the exact behavior performance expected of an individual affected by the intervention [33]. In this case, they asked themselves what the target population exposed to the intervention must do in order to engage in consistent condom use. The team’s review of various meta-analyses on the efficacy of condom use interventions indicated that behavioral interventions rather than just educational type interventions have been shown to promote the greatest changes in condom use [34-36]. Therefore, the following performance objectives for the intervention were selected: (1) to plan condom use when having anal intercourse, (2) to negotiate with a partner the use of a condom during anal intercourse, and (3) to choose to avoid anal intercourse without a condom.

#### Determinants of Condom Use

Once the target behavior and performance objectives had been specified, the next step was to identify determinants, that is, the factors found to be associated with the performance of the behavior [37]. The research team undertook a review of available evidence about determinants contributing to condom use. Two researchers from our multidisciplinary team had previously conducted a study examining the determinants of condom use among HIV-positive men having anal sex with HIV-negative men or men of unknown HIV status [38]. Their results indicated that intention (OR 3.13, 95% CI 1.25-7.81) and self-efﬁcacy (OR 3.62, 95% CI 1.40-9.37) were the main predictors of condom use. Self-efﬁcacy was found to interact with intention, thereby moderating the relationship between intention and behavior (OR 20.96, 95% CI 2.90-151.51) [38]. Their findings were similar to those of a meta-analysis of studies on condom use, which indicated that self-efﬁcacy was a strong predictor of intention to use condoms and of condom use, especially among HIV-positive MSM [39]. Additional studies identified intention as a significant predictor of unprotected anal sex among HIV-positive MSM, indicating that HIV-positive MSM usually act on their stated intentions [38,40,41]. Finally, in a meta-analysis examining sexual risk behaviors of people living with HIV, psychological factors such as a low intention to engage in safer sex, lack of confidence in one’s ability to engage in safer sex practices, belief that one has little control over condom use, and lack of communication were also reported as predictors of risky sex among MSM [20]. On the basis of the evidence reviewed, the research team concluded that the intervention should target individuals’ intention and self-efficacy in order to increase condom use among HIV-positive MSM. Table 1 provides an example of the matrices with one of the performance objectives, which is to plan condom use when having anal intercourse, aligned with the change objectives of intention and self-efficacy (see Table 1).

**Table 1:** Intervention matrices.

Performance objectives (POs) for the Individual	Change objectives and personal determinants	
	Intention	Self-efficacy
PO 1: To plan condom use when having anal intercourse	Formulate an action plan to use condoms every time during sexual intercourse: 1. Buy or obtain condoms; carry a condom or keep condoms nearby; 2. Discuss or negotiate the use of condoms before sexual intercourse; communicate intentions to use a condom; convince or persuade partner to use a condom; 3. Make an agreement to use condoms or not have sex; use condoms correctly; maintain using condoms for every act of sexual intercourse. Formulate a coping strategies plan for potential barriers or difficulties in planning to use condoms when having anal intercourse: Step 1: Recognize and list situations or barriers that may lead you to have unprotected anal intercourse (ie, positive emotional feelings for the partner, wishing to please the partner, not having condoms nearby at the time of sex, becoming “caught up in the passion of the moment,” and negative connotations about condom use); Step 2: Appraise the situation or barriers for potential problems; Step 3: Generate coping alternatives such as active coping strategies—active use of cognitive self-guidance, recall of both AIDS fears and safety benefits, and experience in safer sex.	Express confidence in their ability to use condoms during sexual intercourse 1. Confidence in their ability to buy or obtain condoms: Identifies where he can buy condoms, also after shop closing time Identifies appropriate condoms to buy and use Chooses the right condom size and brand and types of lube 2. Confidence in their ability to always have a condom with them: Describes where he puts away his condoms, to have them available always Describes how he plans to carry a condom always 3. Confidence in their action plan to use a condom: Describes a plan how he takes a condom out at the right moment Demonstrates how to use condoms correctly 4. Ability to identify situations where they feel they may lack confidence in their ability to use condoms: Able to identify and manage situations where they may lack confidence in their ability to use condoms and devise a plan for each situation identified (eg, influence of alcohol andor drugs, being sexually excited or turned on, experiencing pressure to engage in unprotected anal sex from partner).

### Intervention Mapping Step 3: Theory-Based Methods and Practical Strategies

The third step in intervention mapping consists of determining which theories and theoretically based methods would be most effective in achieving the intervention’s performance objectives and then deciding which practical strategies would best operationalize those theoretical methods [18].

A review of the HIV prevention literature demonstrated that interventions informed by a theory were more successful than those that were not [42]. A systematic review indicated that extensive use of theory in an intervention’s design and evaluation was associated with high effect size estimates (*P*=.049) [43]. Theory informs the development of interventions by identifying theoretical constructs to be targeted and by explaining the mechanisms underlying specific behavioral change techniques [43], which increases the likelihood of their positive impact on behavioral change.

When examining various behavioral theories that addressed self-efficacy and intention, 2 particular theories emerged as relevant: the Theory of Planned Behavior and Social Cognitive Theory. Interventions based on these theories were found to be successful in increasing individuals’ self-efficacy and intention to use condoms [44,45]. The Theory of Planned Behavior proposes that intention plays a key role in the prediction of behavior [46]. According to a meta-analysis, interventions based on this theory tended to have moderate to large effects on behavior (*d*=0.36-0.66) [43,46]. Similarly, in a meta-analytical study examining the theory in the context of condom use, intention and planned behavior construct accounted for 12.4% of the variance in condom use behavior [47].

Social Cognitive Theory, which is one of the most widely used models in studies of sexual transmission risk behaviors, identifies self-efficacy as a key determinant of behavior [48]. Specifically, the theory proposes that effective health behaviors are more likely to be adopted when individuals believe that they can implement them [49]. This proposition was supported by a meta-analysis that showed a positive correlation between self-efficacy and condom use [39].

Upon identifying the relevant theories to be used in the development of the intervention, possible theoretical methods and practical strategies to use in the intervention were then identified in addition to considerations to the theoretical parameters; the conditions under which the methods will work.

The 3 approaches that guided this process were to review the literature, engage in discussions with the clinicians in our multidisciplinary team, and consult with the target population. The empirical literature was reviewed to examine the types of theoretical methods and strategies included in interventions found to be effective in increasing condom use among the target population. One component of effective interventions was the provision of facts and information on HIV and STDs, which was expected to change cognitive factors such as attitudes and beliefs. Another targeted technical skills, such as the correct use of condoms, and interpersonal skills, such as those related to negotiating safer sex and assertiveness. The most common methods used to increase self-efficacy and intention involved modeling, role-playing, guided practice, building skills for resistance to social pressure and shifting focus, and enhancing positive social supports. These theoretical methods were derived from both the Social Cognitive Theory and the Theory of Planned Behavior [33,50,51].

Meetings were also held with the local sexual health community clinicians on our team to determine from their perspective which methods and strategies would work in a Web-based intervention. They were asked about methods and strategies currently used in their clinics that they found to be effective to promote condom use within the HIV-positive MSM population. They highlighted the following methods as being of possible use in a Web-based intervention: knowledge acquisition, skills training, problem solving, and persuasive communication.

A focus group of 10 HIV-positive MSM was also conducted to explore their perspectives on acceptable methods and strategies for a Web-based intervention. After they were given information on the intended intervention’s objectives and the theoretical methods and strategies under consideration, they identified the method of using role models and skill building as methods that would interest potential participants. Strategies in implementing the methods included modeling scenarios, interactive activities, skills training, and problem solving. When discussing the possible methods, the parameters of the methods were also discussed with the focus group. The parameters are the conditions under which the methods will work. For example, when discussing the method of “modeling,” some of the parameters include the learner identifying with the model, the model demonstrating feasible skills, model receives reinforcement, and learner perceives a coping model. Discussion with the focus group included the types of individuals whom the target population would identify with. The focus group identified that “peers” would best suit the modeling method. Throughout the intervention, videos using HIV-positive MSM were used. Also, throughout the intervention the models demonstrated their development of an action plan to use condoms and negotiating skills in relation to condom use with their partners. When demonstrating their negotiating skills, various scenarios were demonstrated in order to provide various coping strategies to the possible responses regarding the use of condoms by their partners (see Table 2).

**Table 2:** Selected examples of determinants, theoretical methods, and practical strategies.

Determinants	Theoretical method	Strategy
Intention	Mastery of skill building, modeling resistance, training refusal skills	Real video of individuals speaking about their action plans to always have a condom with them Scenario of two individuals negotiating condom use and the coping strategies used when partner refuses to use a condom
Self-efficacy	Active learning, modeling, guided practice	Character video of individuals negotiating condom use Interactive activity where individuals present responses to common phrases used in negotiating condom use

Focus group participants also noted the importance of having the information tailored to their individual needs and offering the interventions in a peer-to-peer format.

Coincidently, tailoring has been recognized as an important component associated with the development and evaluation of Web-based interventions [52]. Tailoring has been defined as a process of creating individualized communications by gathering and assessing personal data related to a given health outcome to determine what information or strategies would be most appropriate to include to meet an individual’s unique needs [53]. Compared with generic information, tailored feedback messages are more effective because they are more likely to be heard, read, or remembered and viewed as being personally relevant. They foster an active approach to the learning process, engage individuals in building their intention and self-efficacy, and alter their health risk behaviors [54-58]. Web-based interventions offer the possibility of using a constructed computer algorithm to tailor information supplied to participants based on their needs [52]. Delivering the tailored feedback through this Web-based method provides the ability to toggle between modalities, which enhances the user’s experience and understanding of the material [54]. After reviewing the evidence gathered through the 3 previously discussed approaches, the multidisciplinary team confirmed that the Internet would be a viable mode to deliver the intervention.

### Intervention Mapping Step 4

#### Intervention Components and Materials

**Figure 2:**
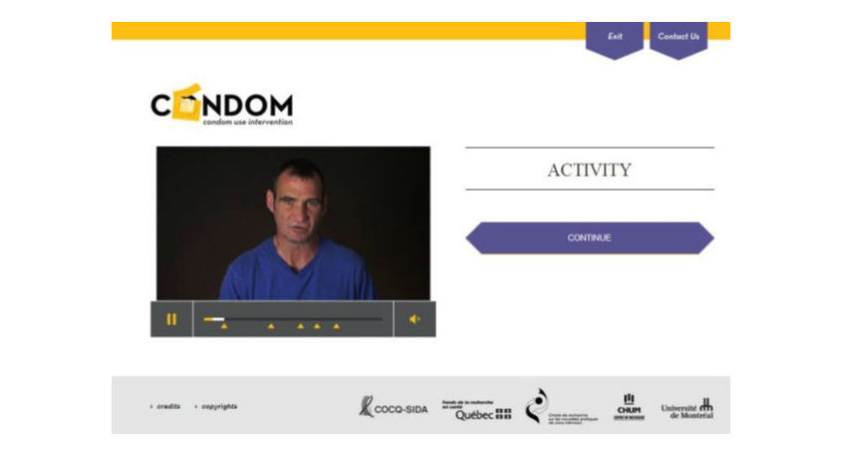
Peer-to-peer tailored video message of a peer’s experiences with consistent condom use represents the method of using role models.

**Figure 3:**
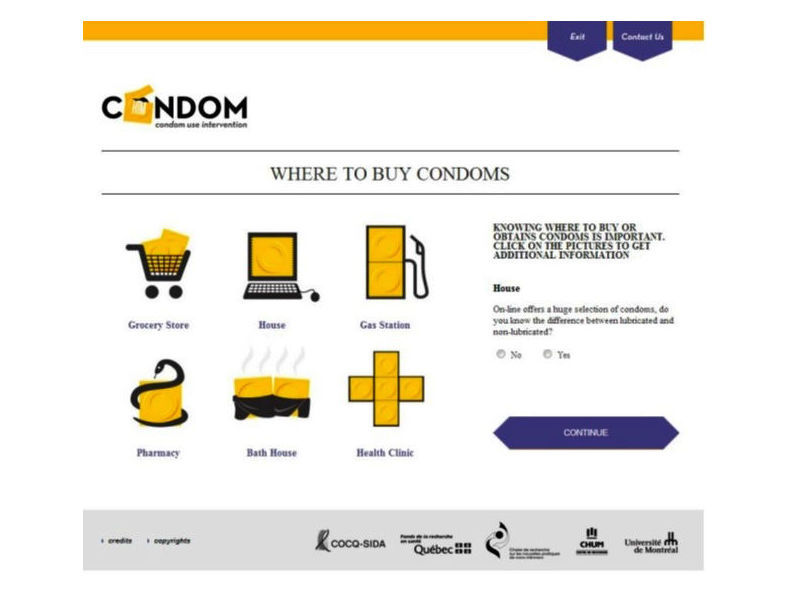
Interactive activity component of the Web-based intervention that facilitates knowledge acquisition.

Step 4 of intervention mapping entails determining the scope and sequence of the intervention’s components and producing the materials for them [18]. Data from the focus group’s perspectives regarding acceptable activities, the kind of visuals to be used in the intervention, and the type of language that would most resonate with our target population were all important factors that the team took into account when developing materials for the Web-based intervention. For example, with regard to making the visual aspect of the intervention attractive to the target population, the focus group participants stressed that it should contain vibrant colors, be interactive, and engage users with pictures of real individuals as well as animated characters. The focus group conceptualized the intervention as representing a journey in which participants would be able to gather information, engage in interactive activities, and receive tailored intervention messages based on their specific needs. They also highlighted the importance of having a peer who would guide users through the intervention and indicated that the language used in the intervention should remain neutral, in order to refrain from offending anyone. Thus, any crude or vulgar terms were to be avoided. Meetings with the Web design team took place to incorporate these aspects from the focus group’s feedback into the design of the intervention (see Figures 2 and 3 for example).

Focus group participants also pointed to the need to include discussions of coping strategies as part of the intervention. They raised issues with previous interventions that gave information and strategies for condom use but neglected to discuss coping strategies to persuade partners to use a condom. A review of the literature and consultation with the health behavior expert on the multidisciplinary team identified a related gap between intention and behavior. Although intentions are considered the best predictors of behavior within the Theory of Planned Behavior, Sheeran’s [59] meta-analysis has shown that intentions alone are not sufficient to predict behavioral change as there are large amounts of behavioral variance that are unexplained. This unexplained phenomenon, which has been termed *the intention to behavior gap* [60], occurs mainly among individuals who have intentions but who fail to act on them [60].

Bridging the gap between intention and behavior could be achieved by means of self-regulation skills, which are crucial for the uptake and maintenance of intended behavioral changes. Planning has been suggested as a self-regulatory skill that mediates between intention and behavior [60-62]. Two types of self-regulatory planning are identified in the literature. Action planning, that is, plans regarding the when, where, and how of implementing the intended behavior, links the behavioral responses to situational cues, thereby facilitating initiation of the intended behavior [60]. Coping planning concerns anticipating the difficulties or barriers that may impede one’s behavioral intentions. It involves making a detailed plan about how to pursue a behavior in the face of obstacles and represents a mental link between anticipated risk situations (ie, situations that endanger the performance of the intended behavior) and suitable coping responses [60,62,63]. Thus, based on the focus group’s recommendations, the review of the literature, and discussions with the team’s health behavior expert, a component was added to the intervention that consisted of action planning and coping planning. As a result, the final intervention is composed of 3 sessions, each lasting approximately 60 minutes, and participants have a total of 2 weeks to complete all 3 sessions. The first session will focus on planning condom use when having anal intercourse; session 2 will focus on negotiating the use of a condom with a partner; and the final session will focus on choosing not to have sexual intercourse without a condom. Each of the 3 sessions is composed of various activities in order to support the individual in accomplishing the session objectives (see Table 3).

**Table 3:** Intervention components.

Session	Objectives	Activities
Session one: 60 minutes	To plan condom use when having anal intercourse	Formulate an action plan to use condoms every time during sexual intercourse Express confidence in their ability to use condoms during sexual intercourse Confidence in their ability to buy or obtain condoms Confidence in their ability to always have a condom with them Confidence in their action plan to use a condom Confidence in their ability to use a condom when either not expected by the partner or there is pressure from partner to not use a condom Ability to identify situations where they feel they may lack confidence in their ability to use condoms adequately (eg, influence of alcohol andor drugs).
Session two: 60 minutes	Negotiate with partner the use of a condom during sexual intercourse	Formulate an action plan to communicate your intentions of using a condom Express confidence in their ability to negotiate or discuss with their partners the use of a condom during anal intercourse Express confidence in their ability to convince partner to use a condom.
Session three: 60 minutes	Choosing not to have sexual intercourse without a condom	Formulate an action plan to refuse to have sex without a condom Express confidence in their ability to refuse having sexual intercourse without a condom Express confidence in their ability to refrain from unprotected sexual intercourse even if decision results in loss of their partner.

#### Tailoring the Intervention’s Components

Once the team selected what kind of components to include in the intervention, deciding on the sequence through which the intervention would be delivered, that is, tailoring, was the next step. The first task in generating an algorithm to tailor the intervention was to determine the process by which the intervention’s messages would be tailored to the participants. Within the literature, message tailoring has been defined as a means of creating communications in which information about individuals is used to determine what specific content they will receive [64]. Hawkins et al [64] identified 3 distinct strategies through which tailoring goals can be achieved: personalization, feedback, and content matching. Discussions were held with the multidisciplinary team in order to debate the most appropriate method for tailoring our intervention.

A decision was made to tailor the intervention’s messages by using a 3-stage approach that incorporated each of the 3 strategies identified by Hawkins et al [64]. The first stage of this process involved assessing participants’ intention and self-efficacy to use condoms. The total score on the included measures would be used to assist in categorizing participants into 4 possible profiles (high intention and high self-efficacy, low intention and low self-efficacy, high intention and low self-efficacy, and low intention and high self-efficacy).

The second stage was based on participants’ individual responses to each of the 12 items on the self-efficacy questionnaire and the 3 items on the intention questionnaire. Participants would be categorized into either a high or a low self-efficacy and intention category according to their response to each item on the questionnaires. The rationale for including this stage was that individuals who had been categorized as either low or high in intention and self-efficacy based on the total sum scores may not mimic the same categorization for each item of the questionnaires. For example, individuals who were categorized as having a high self-efficacy overall could possibly score themselves as having a low self-efficacy in a specific item. This second stage in the approach to tailoring the messages, therefore, would act as a safeguard in addressing specific item responses to the questionnaires that would not be apparent in the overall total sum scoring used in stage 1 of the tailoring process.

All 3 strategies identified by Hawkins et al [64] informed the third stage in our approach toward tailoring the intervention. In it, participants would be given a personalized message intended to validate whether or not they had been categorized correctly according to their responses to the questionnaires. Their response would determine the content of the message they would receive next. Multimedia Appendix 1 presents an excerpt from the intervention algorithm of the 3-stage intervention message tailoring procedure.

After the algorithm for the tailored Web-based intervention messages was completed, a meeting with the Web design team and computer programmer was initiated in order to develop the front end and back end of the intervention. The front end concerned the visual and graphic nature of the Web-based intervention, while the back end concerned the programming component.

### Intervention Mapping Step 5: Adoption, Implementation, and Maintenance

Although step 5 of intervention mapping is focused on the intervention’s adoption, implementation, and maintenance, which must be considered to ensure that the intervention is delivered at acceptable levels of completeness and fidelity, considerations for program implementation actually begin as early as the needs assessment. Throughout the development process the research team did keep in mind the implementation of the intervention.

In addition to considerations for implementation, step 5 in intervention mapping also requires that a detailed plan be drawn up that would influence the behavior of individuals or groups who will make decisions about adopting and using the intervention. Before our intervention goes live on the Internet, both our Web design team and our computer programmer will initiate trial runs with it to test its functioning and implementation over the Internet. The maintenance of the Web-based intervention is a collaborative responsibility of the principal investigator and computer programmer. The computer-tailored Web-based intervention will be housed on the research center’s servers. All data collection and intervention maintenance will be conducted by the principal investigator of the study and the computer programmer, both of whom have access to the intervention.

### Intervention Mapping Step 6: Evaluation Planning

Step 6 of intervention mapping involves designing a plan to evaluate either the efficacy or the effectiveness of the intervention. This plan should be taken into consideration from the beginning of the needs assessment for the intervention and developed along with the rest of the intervention map. A pilot randomized controlled trial is planned to examine the feasibility, acceptability, and preliminary efficacy of this tailored Web-based intervention [65]. Subsequently, the research team will meet to discuss plans to make any adjustments needed in the intervention based on the results of the pilot study. After any adjustments are made, a full-scale, randomized controlled trial is planned in order to evaluate the effectiveness of the Web-based intervention in increasing condom use among HIV-positive MSM.

## Discussion

### Principal Findings

Reporting on the process used to design a particular intervention can serve as a valuable guide for the development of other new interventions and the refinement or revision of existing ones. It also provides a framework and methodology upon which others can build, as well as increases the transparency of the development process and enhances the interpretation of the particular intervention’s effects. In this study, intervention mapping provided the research team with a systematic approach toward developing a theory- and evidence-based health prevention intervention. It afforded guidelines and tools that helped select the theoretical foundations for the intervention and apply the theory in the actual materials and activities produced for the intervention. In addition, the intervention mapping approach allowed for the inclusion of individuals from the target population and clinicians from the field in the development process. The focus group from the target population assisted in validating the methods and strategies developed for the Web-based intervention and was also useful in articulating the visual aspect of the intervention.

The innovations of this particular intervention are its implementation over the Internet and the tailoring process used to assemble its Web-based messages. Given the association between HIV or STD transmission and the high level of Internet use by MSM, the Internet represents a logical method to deliver an intervention to this population. A Web-based intervention allows for the delivery of low-cost prevention messages to a greater number of people, in addition to connecting with individuals who are currently not being reached through more traditional methods [10]. Using the Internet to deliver prevention interventions is still in its infancy stage. To date, very few interventions are delivered completely over the Internet. The majority of them rely on traditional face-to-face interactions and paper-pencil assessments to recruit participants and collect data. This particular Web-based intervention relies solely on the Internet to recruit participants, collect data, and deliver the intervention. Providing a fully Web-based intervention increases efficiency as it involves low costs and has the potential to reach a larger population.

The 3-stage process used to tailor the Web-based messages for this intervention represents another advance in computer tailoring, which has become an increasingly common strategy to alter health risk behaviors [55]. Our tailoring process involves using an electronic algorithm developed by the researchers. Yet, to date, no other study has implemented a participant validation strategy within the tailoring algorithm.

Using intervention mapping to develop a tailored Web-based intervention turned out to be a lengthy and time-consuming process in our case. The main challenges encountered related to the iterative process and working within a large multidisciplinary team. Each development decision made was discussed with members of the research team. This process led to multiple revisions of the intervention. Yet, although time consuming, this process resulted in the production of a concrete intervention that will aim to increase condom use in HIV-positive MSM.

### Conclusions

This paper describes the development process and key components of the Condom-HIM Web-based intervention using intervention mapping. Although a tedious process, the systematic process contributed to the development of a tailored, Web-based theory-driven intervention that has the potential to reach a larger population with minimal human resource costs associated with its implementation and evaluation.

## Appendix

Multimedia Appendix 1 Tailored messaging.

## Abbreviations

HIV: human immunodeficiency virus
MSM: men who have sex with men
OR: odds ratio
STD: sexually transmitted disease
