# Supplementary material for: The Use of Intervention Mapping to Develop a Tailored Web-Based Intervention, Condom-HIM
Source: JMIR Public Health Surveill. 2017 Apr 19;3(2):e20. doi: 10.2196/publichealth.7052 (PMC5415661; doi:10.2196/publichealth.7052)
Supplement: Multimedia Appendix 1 [file publichealth_v3i2e20_app1.pdf]

Animated Character: “Here we are again at the main area of the village. From here we will focus on your intentions to use condoms. Having the intention, to use a condom every time is essential. Your profile shows that overall you may need a little help increasing your intention to use condoms every time. Having a plan in place will help you to regularly use condoms. This plan should include knowing where to buy and obtain condoms; negotiate condom use and making an agreement to not have sex without a condom. Do you have an action plan that includes all of these items?”

If answered yes reinforcement message given:

“This is great. You can never be too ready. Having a plan in place for condom use increases your likelihood of using a condom when the situation arises”

If answered no intervention message given:

“Maybe this is a good time to review some of these topics so that you can add them to your plan. I have a few friends that could each tell you about their plan for condom use. Maybe after listening to some of my friends you might want to use some of their strategies or come up with a few of your own”

Interactive intervention Activity:
Animated Guide: “These are a few of my friends who all have plans in place to help them with consistent condom use. Each one can tell you about their plan, click on each one to learn more about their plans”

(Participant is able to click on each person to hear their message)

1st Friend - Dave: “Hi, my name is Dave and my plan is actually very easy. I always have a couple of condoms and some lube packs on me. They come in convenient travel packs. I keep them in my bag so they are always around. I also keep some in my coat pocket. When I go out, I always grab some and keep them on me. You never know what the evening can bring”.

2nd friend - Steve: “Ever since the time I met a hot guy after last call and realized neither one of us had a condom, I make sure I know where to find some, even after closing hours. Now I make sure I pick up a condom or two when I get to the bar from the free distribution bins, I also ask for a condom when I get my towel and keys at the bathhouse. When I am out late, I know I can find condoms at all night pharmacies and at convenience stores. I will not miss my chance with a hot guy any more. I also make sure I have condoms at home. I can get them for free at the sexual health clinic, at community organizations and through other sources. I also like buying my own, that way I can chose the brand and type of condom I like. No need to be shy, you can meet cute guys in the condom aisle in the pharmacy. Also you can order them online”.

Note: the tailored information presented is for participants profiled as having an overall low intention to use condoms and low intention on having a plan for condom use.
